# A 30‐Year Clinical and Magnetic Resonance Imaging Observational Study of Multiple Sclerosis and Clinically Isolated Syndromes

**DOI:** 10.1002/ana.25637

**Published:** 2019-11-22

**Authors:** Karen K. Chung, Daniel Altmann, Frederik Barkhof, Katherine Miszkiel, Peter A. Brex, Jonathan O'Riordan, Michael Ebner, Ferran Prados, M. Jorge Cardoso, Tom Vercauteren, Sebastien Ourselin, Alan Thompson, Olga Ciccarelli, Declan T. Chard

**Affiliations:** ^1^ Nuclear Magnetic Resonance Research Unit, Queen Square Multiple Sclerosis Centre University College London Institute of Neurology London United Kingdom; ^2^ Medical Statistics Department London School of Hygiene and Tropical Medicine London United Kingdom; ^3^ Department of Radiology and Nuclear Medicine Vrije University, University Medical Center Amsterdam the Netherlands; ^4^ Department of Medical Physics and Biomedical Engineering Centre for Medical Image Computing, University College London London United Kingdom; ^5^ National Institute for Health Research University College London Hospitals, Biomedical Research Centre London United Kingdom; ^6^ Lysholm Department of Neuroradiology National Hospital of Neurology and Neurosurgery London United Kingdom; ^7^ King's College Hospital National Health Service Foundation Trust London United Kingdom; ^8^ Tayside Multiple Sclerosis Research Unit Ninewells Hospital Dundee United Kingdom; ^9^ Wellcome/Engineering and Physical Sciences Research Council Centre for Interventional and Surgical Sciences University College London London United Kingdom; ^10^ School of Biomedical Engineering and Imaging Sciences, King's College London London United Kingdom; ^11^ e‐Health Centre, Open University of Catalonia Barcelona Spain

## Abstract

**Objective:**

Clinical outcomes in multiple sclerosis (MS) are highly variable. We aim to determine the long‐term clinical outcomes in MS, and to identify early prognostic features of these outcomes.

**Methods:**

One hundred thirty‐two people presenting with a clinically isolated syndrome were prospectively recruited between 1984 and 1987, and followed up clinically and radiologically 1, 5, 10, 14, 20, and now 30 years later. All available notes and magnetic resonance imaging scans were reviewed, and MS was defined according to the 2010 McDonald criteria.

**Results:**

Clinical outcome data were obtained in 120 participants at 30 years. Eighty were known to have developed MS by 30 years. Expanded Disability Status Scale (EDSS) scores were available in 107 participants, of whom 77 had MS; 32 (42%) remained fully ambulatory (EDSS scores ≤3.5), all of whom had relapsing–remitting MS (RRMS), 3 (4%) had RRMS and EDSS scores >3.5, 26 (34%) had secondary progressive MS (all had EDSS scores >3.5), and MS contributed to death in 16 (20%). Of those with MS, 11 received disease‐modifying therapy. The strongest early predictors (within 5 years of presentation) of secondary progressive MS at 30 years were presence of baseline infratentorial lesions and deep white matter lesions at 1 year.

**Interpretation:**

Thirty years after onset, in a largely untreated cohort, there was a divergence of MS outcomes; some people accrued substantial disability early on, whereas others ran a more favorable long‐term course. These outcomes could, in part, be predicted by radiological findings from within 1 year of first presentation. ANN NEUROL 2020;87:63–74

Multiple sclerosis (MS) is a highly variable condition. Some people with MS accrue little or no neurological disability over decades,^1^, ^2^ whereas others have their life significantly shortened.^3^ With a view to preventing long‐term disability, there is growing interest in the early use of MS disease‐modifying therapies (DMTs) capable of inducing sustained remission, albeit with the caveat that these may themselves be associated with life‐changing side effects.^4^, ^5^ Given this, it is important that the approach to DMTs should, as far as possible, involve a personalized risk–benefit analysis, ideally early in the disease course.

About 85% of people with MS initially develop the relapsing–remitting form, and first present with a clinically isolated syndrome (CIS), an episode of neurological symptoms that at least partially resolves.^6^ Features associated with more favorable longer‐term outcomes in MS include an early age at symptom onset, an initially relapsing–remitting multiple sclerosis (RRMS) course, optic neuritis (ON), or a predominantly sensory CIS, complete remission after a CIS, and a longer interval between the first and second relapse.^1^, ^7^ Following a CIS, a higher initial brain lesion load and greater accrual of lesions over the first 5 years, measured on magnetic resonance imaging (MRI) scans, are associated with an increased likelihood of developing disability within 20 years.^8^ Lesions within the brainstem and spinal cord also appear to be associated with a greater risk of subsequent disability.^9^, ^10^ Natural history studies have demonstrated that in the majority of people with RRMS, it took over a decade for their mobility to become limited, and over 2 decades before they were immobile without aids.^7^ Given this, assessment of the relationships between early prognostic features and later outcomes ideally requires clinical follow‐up of 2 decades or more.

In this study, we considered 2 main questions: How diverse are clinical outcomes at 30 years following a CIS? Can we identify early on (within 5 years) those who will develop progressive MS or have their life shortened by MS? We addressed these questions using data from a unique cohort of people recruited prospectively following a CIS between 1984 and 1987.^11^, ^12^ The group was followed up clinically and had MRI at 1, 5, 10, 14, 20, and now 30 years. As recruitment predated the DMT era, the cohort was largely untreated.

## Patients and Methods

### *Participants*

One hundred forty people with a CIS were prospectively recruited between 1984 and 1987 at the National Hospital of Neurology and Neurosurgery, and Moorfields Eye Hospital. Eight were subsequently found to have alternative diagnoses.^7^ The cohort has previously been followed up on 5 occasions since their baseline assessment. Participants underwent clinical assessment and an MRI brain scan at baseline, with subsequent follow‐up at 1, 5, 10, 14, and 20 years.^8^, ^11^, ^12^, ^13^, ^14^, ^15^ This is an updated 30‐year follow‐up of the cohort. At 1 year, radiological data without clinical data were obtained; at all other time points both were acquired. The numbers of participants and their demographic characteristics at each time point are detailed in Table 1. CISs were classified as being an ON, transverse myelitis (TM), or brainstem syndrome based on clinical features.

**Table 1:** Demographic Characteristics, Clinical Classification, and Data Availability at Each Follow‐up Time Point

	Follow‐up, yr						
	0	1	5	10	14	20	30
Participants assessed at each time point, n	132	108	94	80	68	104	91
Of those assessed							
Mean age at presentation, yr	32^a^	32	31	32	32	32	30
Female, n (%)	80 (61)	67 (62)	53 (56)	53 (66)	47 (69)	69 (66)	59 (65)
Optic neuritis, n (%)	69 (52)	52 (48)	46 (49)	43 (54)	35 (51)	53 (51)	49 (54)
Transverse myelitis, n (%)	36 (27)	31 (29)	27 (29)	25 (31)	23 (34)	29 (28)	27 (30)
Brainstem syndrome, n (%)	27 (20)	25 (23)	21 (22)	12 (15)	10 (15)	22 (21)	15 (16)
Deaths, n			3	5	7	10	29
Of those deceased							
Death related to MS, n				1	3	3	16
MS, death not related, n							2
MS, undetermined cause, n							1
CIS, death unrelated to MS, n			3	4	4	7	10
Of those known to be alive							
CIS, n	132		44	27	17	30	30
RRMS, MRI, n	0		3	2	2	6	5
RRMS, clinical, n	0		44	40	37	43	30
SPMS, n	0		3	11	12	25	26
Total MRI brain scans, n	132	108	91	66	55	77	63
MRI scan availability, n							
Digital scans	42	0	48	63	55	77	63
Printed scans	61	95	38	3	0	0	0
Scans missing	29^b^	13^c^	5^d^	0	0	0	0
Total EDSS assessment, n, excluding deaths related to MS	118^e^	NA	94	80	68	104	91
Telephone EDSS assessment, n	0	0	0	0	11	27	25

a Based on 127 patients; CIS onset date was not available in 4 individuals, all of whom were subsequently lost to follow‐up.

b Historical lesion count data available in 16 participants.

c Historical lesion count available in 9 participants.

d Historical lesion count available in all 5 participants.

e Determined retrospectively.

CIS = clinically isolated syndrome; EDSS = Expanded Disability Status Scale; MRI = magnetic resonance imaging; MS = multiple sclerosis; NA = not available; RRMS = relapsing–remitting multiple sclerosis.

This study was approved by our institutional ethics committee and the National Research Ethics Service (15/LO/0650). All participants gave informed consent, written if they attended in person, or verbal if they provided clinical information by telephone only. For the deceased members of the cohort, death certificates were obtained where possible (27 death certificates obtained out of 29).

### *Clinical Assessment*

Expanded Disability Status Scale (EDSS)^16^ scores were used to measure disability, retrospectively from notes and participant recall at baseline and at nadir, where clinical improvement had plateaued or at 1 year, whichever was earlier, and prospectively by examination or by telephone^17^ at later time points. Baseline EDSS scores could not be determined in 14 participants due to the absence of notes and unclear recall. In participants not assessed at a given time point, EDSS scores were determined retrospectively from later records and scores from adjacent time points. At 30 years, the Paced Auditory Serial Addition Test (PASAT), an assessment of information processing speed, and Brief International Cognitive Assessment for MS (BICAMS) scores were also obtained for those who attended for review.^18^, ^19^ The BICAMS has 3 components: the Brief Visuospatial Memory Test–Revised (BVMTR), the Symbol Digit Modalities Test (SDMT), and the California Verbal Learning Test (CVLT).

### *Magnetic Resonance Imaging*

**Figure 1:**
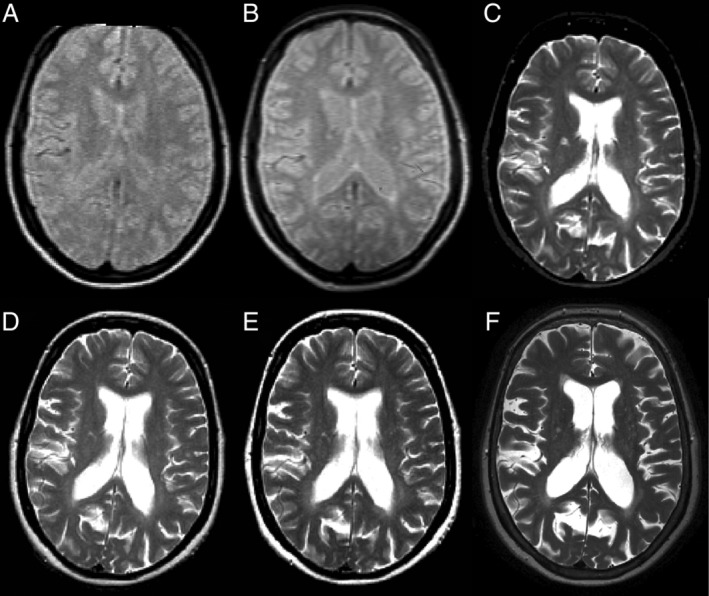
Representative axial brain images from 1 participant, acquired at baseline (A), 5 years (B), 10 years (C), 14 years (D), 20 years (E), and 30 years (F). At baseline and 5 years only proton‐density–weighted images were acquired, whereas at later time points T2‐weighted images were acquired and are shown. Please refer to the main text for a description of the scan acquisition parameters at each time point.

At baseline, 1, and 5 years, scans were acquired using a Picker 0.5T system (Marconi Medical Systems, Cleveland, OH); at 10, 14, and 20 years, a 1.5T General Electric Signa (GE Healthcare, Chicago, IL); at 30 years, a 3T Philips Achieva (Philips Healthcare, Best, the Netherlands). Proton‐density and/or T2‐weighted scans were obtained at each time point. Contiguous, axial slices were obtained, with slice thickness 5 or 10mm at baseline, 5mm at all other time points, and 3mm at 30 years. At baseline, 1, and 5 years, in‐ plane resolution was 1.2 × 1.2mm, repetition time (TR) 2,000 milliseconds, and echo time (TE) 60 milliseconds. At 10, 14, and 20 years, in‐plane resolution was 1.0 × 1.0mm, TR 2,000 milliseconds, and TE 30/90 milliseconds, 14/98 milliseconds, and 17/102 milliseconds, respectively. At 30‐years, in‐plane resolution was 0.5 × 0.5mm, TR 4,375 milliseconds, and TE 85 milliseconds. Figure 1 shows representative MRI scans from each time point.

### *Clinical Outcomes*

Participants were classified as having either a CIS or MS based on the McDonald 2010 criteria.^20^ Those with MS were further subclassified as having this either on clinical (a further relapse or clinical progression) or radiological grounds (new lesions seen on MRI), and RRMS or secondary progressive multiple sclerosis (SPMS).^21^ Death due to MS was determined by consensus review of death certificates or notes (where available) by K.K.C. and D.T.C., where MS was either given as the cause of death or a clear contributing factor to death, for example, aspiration pneumonia in someone with advanced MS or a pulmonary embolus secondary to chronic immobility.

A working definition of nondisabling MS at 30 years was an EDSS score of ≤3.5 (fully ambulatory, with or without abnormal neurological findings on examination).^16^ At 30 years, disease course was classified as CIS, MS with EDSS scores ≤3.5, MS with EDSS scores >3.5, or death relating to MS. Recognizing that the EDSS scores take little account of cognition,^22^ we determined the proportion in each group who were found to have cognitive impairment on the BICAMS. We also determined the proportion who remained in employment or who had retired at the national state pension age of 60 years.

### *MRI Analysis*

Film prints from baseline, 1, 5, and 10 years were redigitized using a VIDAR Diagnostic Pro Advantage film digitizer (VIDAR Systems, Herndon, VA), and processed to reconstruct a digital image stack comparable with native stacks (see Table 1).^23^ For each participant, all available scans were reviewed side by side, using 3D Slicer version 4.4.^24^ White matter (WM) lesions were marked by consensus (K.K.C. with F.B., D.T.C., or both), with reference to preceding or subsequent scans, and then counted by K.K.C. Whole brain, juxtacortical (JC), periventricular (PV), infratentorial (IT), and deep white matter (DWM) lesions were counted separately. DMW lesions were defined as supratentorial lesions that were neither JC nor PV.

### *Statistics*

Early prediction models were fitted from the perspective of earlier time points, when future or final outcomes and diagnostic groups were unknown, and therefore, unless otherwise stated, include all available subjects, including those who remained classified as CIS. Univariate and multivariate logistic regression was used to identify early (baseline, 1 year, and 5 year) predictors of the following 3 binary 30‐year outcomes: (1) 30‐year EDSS scores ≤3.5 versus 30‐year EDSS scores >3.5 (including deaths due to MS [ie, EDSS scores = 10 by 30 years]); this EDSS cutoff was chosen a priori as more clinically meaningful and objective than the >3.0 versus ≤3.0 threshold; (2) SPMS diagnosis by 30 years, including SPMS deaths, versus CIS and RRMS at 30 years; and (3) death due to MS by 30 years versus all still alive at 30 years. Independent variables analyzed are listed in the Results section. Additionally, for MS‐associated death, a Cox proportional hazards model was used to identify the best predictors. All deceased participants, regardless of MS status and cause of death, contributed to the Cox survival analysis, censored at the time of death. Individuals whose deaths were unrelated to MS were not included in the models for 30‐year outcomes. The categories for early EDSS, EDSS changes, and lesion count predictors were categorized to generate approximately equal frequencies; binary lesion variables, where possible, were dichotomized a priori 1+ versus 0 lesions, or to equalize frequencies if 0/1+ resulted in a very unequal distribution. Resulting ordered categorical variables were naturally coded so that when entered into a model, the coefficient gave a linear test for monotonic trend across the increasing category levels, assuming equal steps between adjacent categories. When the ordinal lesion variables did not predict materially better than binary, models with binary lesion predictors were reported. For multivariate logistic and Cox models, manual backward stepwise elimination of variables with *p* > 0.05 was used to identify the best subset of independent predictors. Age‐adjusted comparisons of cognitive outcomes between groups at 30 years were performed using multiple regression of the cognitive measure on group indicators, with age as covariate. Analyses were performed using Stata 15.1 (StataCorp, College Station, TX),^25^ and statistical significance is reported at *p* < 0.05.

## RESULTS

### *Whole Cohort*

At the 30‐year follow‐up, outcome data (including deaths) were obtained in 120 out of the original 132 participants. Twelve individuals declined or were not traceable. Twenty‐nine individuals were deceased, of whom 19 had MS, and 10 died with last known classification as CIS. Of these 10 participants, 3 were last assessed at 20 years, 1 at 10 years, 2 at 5 years, 2 at 1 year, and 2 at baseline. The mean follow‐up duration was 30.9 years. Table 1 summarizes the number of participants with a known outcome at each time point. In those alive at 30 years, the mean (standard) age was 61.6 years (7.4 years), with 59 (65%) female and 32 (35%) male. In the 91 alive individuals, 30 remained classified as having had a CIS, and 61 had MS. In total, 80 were known to have MS (61 alive and 19 deceased). BICAMS scores were obtained in 61 participants, 41 with MS and 20 with CIS. Table 2 shows baseline demographic and clinical features for all participants, based on the 30‐year outcome.

**Table 2:** Baseline Demographic and Clinical Features for All Participants Based on 30‐Year Outcome

	Mean Age at Presentation, yr	Female, n (%)	CIS Presentation (% of each 30‐year outcome, % within each CIS presentation)		
			Optic Neuritis	Brainstem	Transverse Myelitis
Baseline total, N = 132	32	80 (61%)	69	27	36
30‐year outcome					
CIS, n = 30	31	20 (67%)	19 (63, 28)	3 (10, 11)	8 (27, 22)
RRMS EDSS ≤3.5, n = 32	30	20 (63%)	15 (47, 22)	7 (22, 26)	10 (31, 28)
RRMS EDSS >3.5, n = 3	26	2 (67%)	2 (66, 3)	0	1 (33, 3)
SPMS, n = 26	32	17 (65%)	13 (50, 19)	5 (18, 19)	8 (31, 22)
Deceased due to MS, n = 16	36	10 (63%)	7 (44, 10)	6 (38, 22)	3 (19, 8)
Deceased with CIS, n = 10	35	4 (40%)	4 (40, 6)	3 (30,11)	3 (30, 8)
Deceased with MS (not due to MS or unknown), n = 3	40	2 (67%)	1 (33, 1)	2 (67, 7)	0
Unknown outcome, n = 12	31	5 (42%)	8 (67, 12)	1 (8, 4)	3 (3, 8)

CIS = clinically isolated syndrome; EDSS = Expanded Disability Status Scale; MS = multiple sclerosis; RRMS = relapsing–remitting multiple sclerosis; SPMS = secondary progressive multiple sclerosis.

## MS Cohort

Of the 80 people known to have MS by 30 years, 19 were deceased. Sixteen died of complications relating to advanced MS (EDSS scores = 10), 2 died of unrelated causes, and for 1 the cause of death was unknown. All 3 were assessed and documented to have RRMS at 20 years, with EDSS scores of 2.5, 3.0, and 6.0. Of the 61 who were alive, 26 had SPMS and 35 RRMS. BICAMS scores were obtained in 41 (26 with EDSS scores ≤3.5, 15 with EDSS scores >3.5), and BICAMS *z* scores (adjusted for age, sex, and years of education) were available in 31 subjects who were ≤ 65 years of age. Subjects who did not complete the cognitive tests tended to be more physically disabled, both early (mean nadir EDSS scores 1.6 vs 0.8 for subjects who completed assessment) and later in the disease course (mean 30‐year EDSS scores 6.0 vs 4.1). Eleven (14%) have had a DMT at some stage, all of which were first‐line injectable drugs, with the earliest commencing 10 years after MS diagnosis (when DMTs first became available in the United Kingdom). Of these, 7 had SPMS at 30 years, and 4 had RRMS.

**Figure 2:**
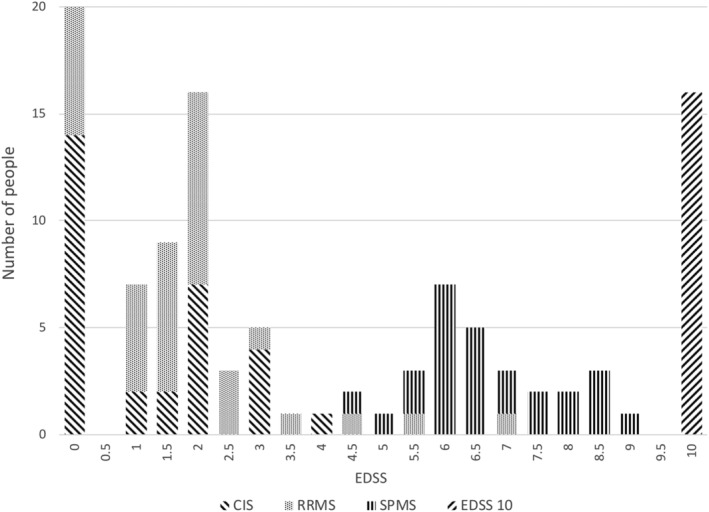
Expanded Disability Status Scale (EDSS) scores at 30 years. EDSS scores were obtained from 107 individuals at 30 years. An EDSS score of 10 was only assigned to those where multiple sclerosis (MS) was known to have contributed to death. In the 3 other people with MS who had died, the cause of death was either unrelated to MS or unknown, and no EDSS score was assigned. CIS = clinically isolated syndrome; RRMS = relapsing–remitting multiple sclerosis; SPMS = secondary progressive multiple sclerosis.

At 30 years, EDSS score peaks were observed at 0, 2.0, 6.0, and 10, with the lowest points at 4.0 and 9.5 (Fig 2). All of the 26 with SPMS (34%) had EDSS scores >3.5. Of the 35 (45%) with RRMS, 32 (42%) had EDSS scores ≤3.5. Six people fulfilled 2010 MS diagnostic criteria on radiological rather than clinical grounds, and they all had EDSS scores ≤3.5.

**Figure 3:**
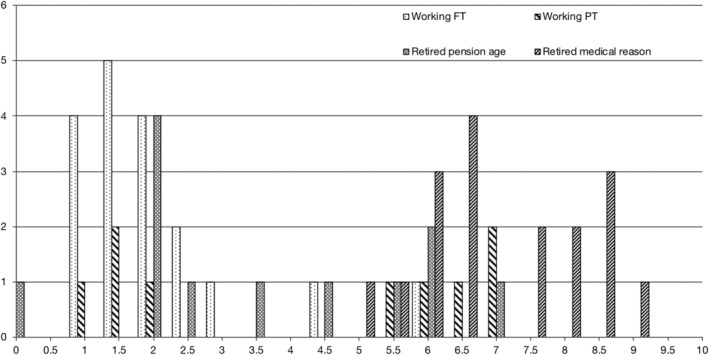
Employment and retirement status in people with multiple sclerosis, by the Expanded Disability Status Scale at 30 years (n = 61). FT = full time; PT = part time.

With regard to cognition, of the 32 with EDSS scores ≤3.5, 21 had validated BICAMS *z* scores, of whom 2 had a *z* score of <−1.5 in 1 or more modalities. None of the 32 had retired early for medical reasons, and all remained in employment (full‐ time or part time), or retired at the national state pension age (Fig 3). Age‐adjusted cognitive measures in the group with MS and EDSS scores ≤3.5 were not significantly different from the CIS group: for the PASAT, the MS with EDSS scores ≤3.5 group (adjusted mean 42.32) was 9% worse than the CIS group (adjusted mean = 46.31, difference = −4.00, *p* = 0.26, 95% confidence interval [CI] = −11.0 to 3.0); for the BVMTR (adjusted mean = 25.89), 0.2% better than CIS (adjusted mean = 25.84, difference = 0.05, *p* = 0.97, 95% CI = −0.03 to 3.4); for the CVLT (adjusted mean = 51.70), 1% worse than CIS (adjusted mean = 52.41, difference = −0.71, *p* = 0.83, 95% CI = −7.3 to 5.8); for the SDMT (adjusted mean = 50.40), 7% worse than CIS (adjusted mean = 54.39, difference = −4.0, *p* = 0.10, 95% CI = −8.7 to 0.7). In the group with MS and EDSS scores >3.5, cognitive measures were more substantially and significantly worse than the CIS group (except for CVLT): for the PASAT, 23% worse (adjusted mean = 35.63, difference = −10.68, *p* = 0.008, 95% CI = −18.4 to −2.9); for the BVMTR, 21% worse (adjusted mean = 20.40, difference = −5.44, *p* = 0.006, 95% CI = −9.3 to −1.6); for the CVLT, 9% worse (adjusted mean 47.92, difference = −4.49, *p* = 0.23, 95% CI = −11.9 to 2.9); and for the SDMT, 24% worse (adjusted mean = 41.49, difference = −12.90, *p* < 0.001, 95% CI = −18.3 to −7.5).

### *Early Predictors of 30‐Year Outcome*

#### *Demographic and Clinical Features as Predictors*

Univariate predictors of 30‐year outcomes are presented in the Supplementary Table. There was no association of gender and disease duration with any of the 30‐year outcome groups. People presenting with a brainstem CIS were at greater risk than those presenting with either ON or TM (of MS‐related death, hazard ratio [HR] = 2.87, *p* = 0.04). This was consistent with the higher proportion of brainstem subjects with baseline IT lesion present (41%), compared to TM (19%) and ON (11%; χ^2^ test, *p* = 0.009). The change in EDSS scores from nadir to 5 years was also largest in the brainstem CIS group (mean = 1). Older people at presentation were at greater risk of MS‐related mortality (HR = 1.07 per year, *p* = 0.04).

#### *EDSS as a Predictor*

**Figure 4:**
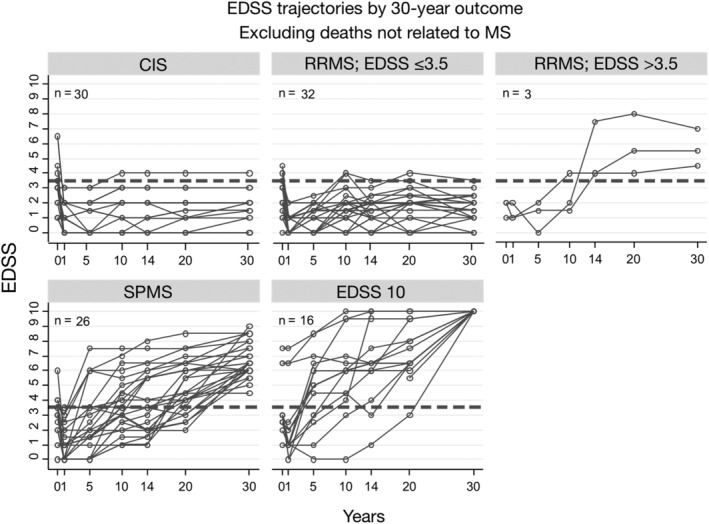
Expanded Disability Status Scale (EDSS) trajectories over 30 years by 30‐year status. CIS = clinically isolated syndrome; MS = multiple sclerosis; RRMS = relapsing–remitting multiple sclerosis; SPMS = secondary progressive multiple sclerosis.

Figure 4 shows the EDSS trajectories over time, based on 30‐year outcome: separation between groups becomes apparent at 5 years, and is more pronounced subsequently. Baseline EDSS scores were not significantly associated with 30‐year outcome.

For prediction of SPMS following a CIS, there was no significant association with either baseline or nadir EDSS scores, but 5‐year EDSS scores ≥2.5 was a significant predictor: 18 of 25 (72%) in this group had developed SPMS by 30 years, compared to 18 of 84 (21%) in the 5‐year EDSS scores <2.5 group, with an odds ratio (OR) of 9.43 (*p* < 0.001, 95% CI = 3.4 to 26.1), positive predictive value (PPV) = 72%, negative predictive value (NPV) = 79%, sensitivity = 50%, specificity = 90%, and accuracy = 77% (95% CI = 68 to 85%). EDSS change from nadir to 5 years was a slightly stronger predictor than either the absolute 5‐year EDSS value, or the change from baseline to 5 years; 13 of 14 (93%) people who progressed by ≥2 EDSS points between nadir and 5 years had transitioned to SPMS, compared to 23 of 95 (24%), OR = 40.7 (*p* < 0.001, 95% CI = 5.0 to 328.2), PPV = 93%, NPV = 76%, sensitivity = 36%, specificity = 99%, and accuracy = 78% (95% CI = 69 to 85%).

For MS‐related death, 5‐year EDSS scores were the strongest early predictor in a survival analysis (there were no MS‐related deaths by 5 years). Five‐year EDSS scores ≥2.5 versus <2.5 gave a HR of 23.6 (*p* < 0.001, 95% CI = 5.3 to 105.9); in those with 5‐year EDSS scores ≥2.5, 12 of 29 (41%) were deceased by 30 years, compared to 2 of 88 (2%) in those with EDSS scores <2.5, PPV = 41%, NPV = 98%, sensitivity = 86%, specificity = 84%, and accuracy = 84% (95% CI = 76 to 90%).

For predicting 30‐year EDSS score ≤ 3.5 versus EDSS score > 3.5 outcome, EDSS scores at nadir and 5 years were significant, more so than EDSS changes between these time points, and the predictive value of EDSS scores at 5 years was, unsurprisingly, greater than at earlier time points.

A greater proportion of people with nadir EDSS scores ≥2.5 progressed to 30‐year EDSS scores >3.5, 11 of 14 (79%) compared to 33 of 91 (36%) in those with nadir EDSS scores <2.5, OR = 6.44 (*p* = 0.007, 95% CI = 1.7 to 24.8), PPV = 79%, NPV = 64%, sensitivity = 25%, specificity = 95%, and accuracy = 66% (95% CI = 56 to 75%); for 5‐year EDSS scores ≥2.5, the corresponding proportions were 57 of 78 (73%) versus 4 of 27 (15%), OR = 15.6 (*p* < 0.001, 95% CI = 4.8 to 50.5), PPV = 85%, NPV = 73%, sensitivity = 52%, specificity = 93%, and accuracy = 76% (95% CI = 67 to 84%).

#### *Lesion Counts and Locations as Predictors*

Total baseline lesion count was a significant predictor (*p* < 0.001) of 30‐year EDSS scores >3.5, with OR = 1.84 (trend test *p* < 0.001, 95% CI = 1.3 to 2.5) per adjacent high lesion number category (0, 1–3, 4–10, 11–20, 21+), or the reciprocal OR, 0.54 for 30‐year EDSS scores ≤3.5. The strongest early MRI predictors of 30‐year EDSS scores >3.5 were the presence of (1) baseline IT lesions, with 19 of 22 (86%) progressing to EDSS scores >3.5 by 30‐years, versus 25 of 74 (34%) of those without baseline IT lesions, OR = 12.4 (*p* < 0.001, 95% CI = 3.4, 46.0), accuracy = 71% (95% CI = 61 to 80%); (2) 1‐year DWM lesions, with 35 of 58 (60%) reaching EDSS scores >3.5 versus 3 of 24 (13%), OR = 10.7 (*p* < 0.001, 95% CI = 2.8 to 39.8), accuracy = 68% (95% CI = 57 to 78%); and (3) 1‐year IT lesions, 20 of 24 (83%) versus 18 of 58 (31%), OR = 11.1 (*p* < 0.001, 95% CI = 3.3 to 37.2), accuracy = 73% (95% CI = 62 to 82%). For predicting 30‐year EDSS scores ≤3.5, these ORs apply to the absence of these lesions.

Given that 91% of those with 30‐year EDSS scores >3.5 had SPMS, the strongest MRI predictors for SPMS were the same: the presence of baseline IT lesion, with 19 of 22 (86%) becoming SPMS, versus 20 of 84 (24%) for those without these lesions, OR = 20.3 (*p* < 0.001, 95% CI = 5.4 to 75.6), accuracy = 78% (95% CI = 69 to 86%); DWM lesions at 1 year, 32 of 62 (52%) versus 2 of 30 (7%), OR = 14.9 (*p* < 0.001, 95% CI = 3.3 to 68.1), accuracy = 65% (95% CI = 55% to 75%); and IT lesions at 1 year, 20 of 24 (83%) versus 14 of 68 (21%), OR = 19.3 (*p* < 0.001, 95% CI = 5.7 to 65.6), and accuracy = 65% (95% CI = 55 to 75%).

For MS‐related mortality, in a survival analysis, presence of baseline IT lesions had an HR of 3.9 (*p* = 0.007, 95% CI = 1.5 to 10.3); the proportion of deaths in those with these lesions was 8 of 23 (35%) versus 8 of 78 (10%) in those without, PPV = 35%, NPV = 90%, sensitivity = 50%, specificity = 82%, and accuracy = 77% (95% CI = 68 to 85%); presence of 1‐year IT lesions had an HR of 5.25 (*p* = 0.003, 95% CI = 1.8 to 15.7), with corresponding proportions 9 of 26 (35%) versus 5 of 61 (8%), PPV = 35%, NPV = 92%, sensitivity = 64%, specificity = 77%, and accuracy = 75% (95% CI = 64 to 83%).

#### *Combined Predictive Models*

Variables entered into multivariate predictive models, to determine best predictors, include: age at onset, gender, CIS type, disease duration, early EDSS scores and interval EDSS changes between time points, number of relapses within the first 5 years; and early total lesion count, changes in total lesion count, and location‐specific lesion counts. Overall, MRI‐detected brain lesions proved more effective predictors of 30‐year outcomes than EDSS: in multivariate models including both lesion and EDSS variables, the latter no longer contributed significantly, with their coefficients substantially reduced.

Tables 3 and 4 show the results for early prediction of 30‐year EDSS scores >3.5 and 30‐year SPMS. For each of the 2 outcomes, 2 models are shown: up to 1 year and up to 5 years. IT and DWM lesions were the best predictors, with the addition of nadir‐to‐5‐years EDSS change in the SPMS prediction model. The up to 1‐year models show that subjects with neither baseline IT nor 1‐year DWM lesions had a 13% probability of 30‐year EDSS scores >3.5 (87% probability of 30‐year EDSS scores ≤3.5), whereas subjects with at least 1 lesion of both types had 94% probability (95% CI = 83 to 100%) of 30‐year EDSS scores >3.5, and 94% probability of SPMS by 30 years. The up‐to‐5‐years models show that subjects with ≤5 DWM lesions at 5 years and EDSS change of <2 from nadir to 5 years, had 11% probability of SPMS by 30‐years; conversely, subjects with >5 DWM lesions and ≥ 2 EDSS change had 96% probability (95% CI = 86 to 100%) of SPMS.

**Table 3:** Best Independent Early Predictors of 30‐Year EDSS >3.5 and ≤3.5. All models include EDSS 10 at or before 30 years.

Predictor	Odds Ratio (95% CI)	*p*	Predictor Combinations			
**30‐year EDSS >3.5, best independent predictors up to 1 year** a						
Baseline infratentorial lesion count, ≥1 vs 0	16.8 (2.0–139.7)	0.009	0	0	≥1	≥1
1‐year deep white matter lesion count, ≥1 vs 0	6.7 (1.7–26.0)	0.006	0	≥1	0	≥1
Model‐predicted probabilities for 30‐year EDSS >3.5 (95% CI)			13% (0–26)	49% (33–64)	—b	94% (83–100)
Model‐predicted probabilities for 30‐year EDSS ≤3.5^c^			87%	51%	—	6%
**30‐year EDSS >3.5, best independent predictors up to 5 years** d						
Baseline infratentorial lesion count, ≥1 vs 0	8.0 (1.5–41.4)	0.013	0	0	≥1	≥1
5‐year deep white matter lesion count, >5 vs ≤5	5.1 (1.7–15.6)	0.004	≤5	>5	≤5	>5
Model‐predicted probabilities for 30‐year EDSS >3.5 (95% CI)			18% (5–30)	52% (36–71)	63% (22–100)	90% (76–100)
Model‐predicted probabilities for 30‐year EDSS ≤3.5			82%	48%	37%	10%

All models include EDSS = 10 at or before 30 years.

a Model n = 80. Overall model accuracy using 0.5 probability cutoff (95% CI) = 71% (60–81).

b There were no subjects with this lesion combination.

c These probabilities and their CIs are 100% minus the >3.5 probabilities.

d Model n = 79. Overall model accuracy using 0.5 probability cutoff (95% CI) = 75% (64–84).

CI = confidence interval; EDSS = Expanded Disability Status Scale.

**Table 4:** Best Independent Early Predictors of 30‐Year SPMS Status. All models include EDSS 10 at or before 30 years.

Predictor	Odds Ratio (95% CI)	*p*	Predictor Combinations			
**30‐year SPMS, best independent predictors up to 1 year** a						
Baseline infratentorial lesion count, ≥1 vs 0	26.0 (3.1–215.0)	0.003	0	0	≥1	≥1
1‐year deep white matter lesion count, ≥1 vs 0	8.6 (1.8–41.0)	0.007	0	≥1	0	≥1
Model‐predicted probabilities for 30‐year SPMS (95% CI)			7% (0–16)	38% (23–53)	—	94% (83–100)
**30‐year SPMS, best independent predictors up to 5 years** b						
5‐year deep white matter lesion count, >5 vs ≤5	5.3 (1.7–16.6)	0.005	≤5	≤5	>5	>5
EDSS score change from nadir to 5‐year, ≥2 vs <2	31.1 (3.5–279.9)	0.002	<2	≥2	<2	≥2
Model‐predicted probabilities for 30‐year SPMS (95% CI)			11% (2–21)	80% (46–100)	41% (24–57)	96% (86–100)

All models include EDSS = 10 at or before 30 years.

a Model n = 89. Overall model accuracy using 0.5 probability cutoff (95% CI) = 79% (69–87).

b Model n = 85. Overall model accuracy using 0.5 probability cutoff (95% CI) = 78% (67–86).

CI = confidence interval; EDSS = Expanded Disability Status Scale; SPMS = secondary progressive multiple sclerosis.

For MS‐related death, the best independent early predictors up to 1 year were having ≥1 IT lesions at 1 year, HR of 3.6 (95% CI = 1.1 to 11.2, *p* = 0.031), and nadir EDSS scores, HR of 1.5 per additional EDSS score unit (95% CI = 1.2 to 2.0, *p* = 0.003)

## Discussion

This cohort provides a unique perspective on the long‐term clinical and MRI evolution of relapse‐onset MS. As MRI first became available in the 1980s and DMTs in the 1990s, it is highly unlikely that such long‐term, essentially natural history, data can be obtained again. The results from this study suggest that 30 years following symptom onset, there are 3 distinct MS outcomes: an RRMS group with little accrued disability (EDSS scores ≤3.5), an SPMS group who all had impaired mobility (EDSS scores ≥4.0), and a group who have had their lives shortened by MS (all of whom had SPMS). The results also suggest that, at 30 years, cognitive assessment scores in the EDSS scores ≤3.5 group were not significantly different from the CIS group, whereas in the EDSS scores >3.5 group, they were worse. Thirty‐year outcomes could, in part, be predicted by early EDSS scores and more robustly by MRI‐derived regional lesion counts.

After allowing for other factors, 30‐year outcomes were not independently associated with age at onset, gender, baseline EDSS score, and CIS type. MRI lesion counts proved to be better predictors than EDSS scores, and lesion location was more important than lesion number. There was more missing data for changes in lesion count than for absolute lesion counts, and this may be a factor in why new early lesions were not as predictive. Interestingly, although PV and JC lesions are highly relevant in the diagnosis of MS,^20^ it was early IT and DWM lesions that had the greatest long‐term prognostic value. For example, in people with baseline IT and DWM lesions by 1 year, the chances of having SPMS were 94%, whereas those with 1 or more IT lesions by 1 year were 5 times more likely to have died due to MS than the rest of the cohort. Conversely, absence of both baseline IT and 1‐year DMW lesions gave an 87% probability of EDSS scores ≤3.5 at 30 years. IT lesions have previously been linked with less favorable outcomes in people with MS, after a mean follow‐up of 7.7 years.^9^

Considering the potential application of these results, treatment decisions are often made prospectively, and increasingly early in the disease course, prognostic factors identified within a year of symptoms onset may prove more useful than those identified within 5 years. However, favorable prognostic features at 1 year may also not impact significantly on choices; instead, the emergence of markers suggestive of more disabling outcomes may carry more weight.

Since this cohort was first recruited, diagnostic criteria for MS have changed, and most significantly an MS diagnosis can now be made in people after only a single episode of symptoms, but who fulfil MRI criteria for dissemination of lesions in space and time.^20^ In the present study, 6 individuals had MS diagnosed on radiological grounds (all had EDSS scores ≤3.5 at 30 years, compared with 37% if diagnosed on clinical grounds). Thus, they appear to represent a clinically silent end of the MS spectrum, who would previously have been overlooked. With the routine use of DMTs, the long‐term evolution of MS may be changing: in a large cohort of RRMS patients, where 62% were treated with DMTs, only 11.3% transitioned to SPMS after a 17‐year period.^26^

There are several study limitations. First, this study used well‐established clinical outcome measures. This is less controversial for physically disabling outcomes such as SPMS or MS‐related deaths, but what is considered a nondisabling outcome may differ substantially depending on whose perspective it is from, and patient‐reported outcomes have not been assessed.^27^, ^28^ For example, we have not included detailed assessments of fatigue and visual impairment, which may significantly affect functional outcomes in people with MS. With this in mind, the nondisabling MS group identified in this study may more pragmatically be considered to be people with MS who have consistently low levels of disability with no progression, and less to gain from DMTs, rather than those who have no ill effects from MS. Second, at the inception of this cohort, MRI was a new technique, and image quality was not as good as is achievable now; given this, analyses of the earlier images will be less reliable than later ones. Postgadolinium sequences were not obtained at any time point, and only limited T1‐weighted images were obtained at 14 and 20 years, and as such we have not been able to assess active lesion inflammation at the time of scanning or assess the early relevance of T1 “black holes” for longer‐term outcomes. Third, symptoms attributable to spinal cord involvement were not systematically assessed early on in this cohort, and spinal cord imaging was not routinely obtained. Additionally, we were not able to obtain outcome data for 12 of the 132 original participants, nor were we able to obtain MRI scans in 9 of the 30 participants classified as CIS; it is possible that some of these individuals would fulfil MS diagnostic criteria, although this is unlikely to change the main findings of this study. Twelve participants did not contribute to early MRI information, of whom 7 were lost to 30‐year follow‐up; however, the similarity in their baseline demographic features to the rest of the cohort suggests it is unlikely our main results are materially affected. It should also be noted that the cohort originated from 1 neurosciences center, and therefore there may be limitations in generalizability.

With regard to EDSS data, particularly early in the study, these were not captured consistently. To minimize inaccuracies, data from adjacent time points and from clinical records, where available, were used. However, it is worth noting that EDSS scores ≤3.5 are derived from symptoms and examination findings, whereas scores from 4 upward represent thresholds of mobility impairment. Although a > 3.0 versus ≤3.0 threshold has been proposed in the literature on benign MS, our use of an EDSS threshold of ≤3.5 versus >3.5 at 30 years is more objectively interpretable, should minimize the impact of any inter‐ or intrarater variabilities, and be more reliable in the predictive models. Furthermore, only 1 participant in our sample would have been reclassified if a > 3.0 versus ≤3.0 dichotomy was used, with little impact on the main results. With regard to cognition, it is worth noting that of those who did not complete cognitive assessment, when compared to those who did, a significantly higher proportion were more neurologically disabled. Given this, it is likely that we have underestimated the true magnitude of cognitive deficits in those who are more physically disabled, and it is also possible that the small differences observed between the EDSS scores ≤3.5 and CIS groups have been underestimated due to incomplete data.

In our statistical analyses, as the main focus of our study is on early (within 5 years) predictors of late outcomes (30 years), we have confined ourselves to investigating only the associations between these time points, and not associations at all other time points. Analyses of the intermediate time points would be of interest, but would answer different questions and lie outside the scope of the present article. A further caveat is that some subgroups were small, resulting in estimated odds ratios that, although statistically significant, should be interpreted with caution, particularly where the ORs’ confidence intervals are very wide. Classification properties of our multivariate models might be improved with probability cutoffs different from 0.5; however, we believed this optimization may not be reliably generalizable and preferred not to screen for the best classification.

Lastly, it is worth noting that ON as a presenting CIS has been associated with a more favorable outcome when compared with other presentations.^1^ Fifty‐two percent of the participants in this study presented with ON, whereas in another large prospective cohort study, 37% presented with ON.^29^ Although there was no evidence of association between ON and 30‐year outcome in this study, there may still be some bias toward more favorable outcomes.

In conclusion, the results of this study suggest a divergence of natural outcomes in people with MS 30 years after symptom onset: those with SPMS, who have developed greater disability and have a significant risk of their life being shortened by MS; and those classified as having RRMS, who remained fully ambulatory, with no significant cognitive impairment, and who remained employed or retired at the expected age. The results also indicate that for less favorable outcomes, the die may be cast early. This suggests that there are people with MS who have more to gain from earlier use of higher efficacy DMTs, although also counsels caution when considering the blanket use of DMTs in early MS or following a CIS. The predictive models developed by this study include features that can be obtained in clinical practice, and so hopefully may inform risk–benefit analyses when considering DMTs. A key goal of future research is to determine what pathologically differentiates progressive from persistently nonprogressive MS, with a view to targeting treatments that would substantially increase the chances a person with MS follow a less‐disabling clinical course.

## Author Contributions

D.T.C. contributed to study concept and design. K.K.C., D.A., F.B., K.M., P.A.B., J.O., M.E., F.P., M.J.C., T.V., S.O., and D.T.C. contributed to data acquisition and analysis. K.K.C., D.A., F.B., A.T., O.C., and D.T.C. contributed to drafting of the manuscript and figures.

## Potential Conflicts of Interest

Nothing to report.

## Supporting information


**SUPPLEMENTARY TABLE 1** Early Univariable Predictors of 30‐Year Outcomes

## References

[1] Ramsaransing GSM , De Keyser J. Benign course in multiple sclerosis: a review. Acta Neurol Scand 2006;113:359–369. 16674602 10.1111/j.1600-0404.2006.00637.x

[2] Hawkins SA , Mcdonnell G V . Benign multiple sclerosis? Clinical course, long term follow up, and assessment of prognostic factors. J Neurol Neurosurg Psychiatry 1999;67:148–152. 10406979 10.1136/jnnp.67.2.148 PMC1736487

[3] Scalfari A , Knappertz V , Cutter G , et al. Mortality in patients with multiple sclerosis. Neurology 2013;81:184–192. 23836941 10.1212/WNL.0b013e31829a3388 PMC3770174

[4] Soelberg Sorensen P. Safety concerns and risk management of multiple sclerosis therapies. Acta Neurol Scand 2017;136:168–186. 27891572 10.1111/ane.12712

[5] Auricchio F , Scavone C , Cimmaruta D , et al. Drugs approved for the treatment of multiple sclerosis: review of their safety profile. Expert Opin Drug Saf 2017;16:1359–1371. 28976217 10.1080/14740338.2017.1388371

[6] Miller DH , Chard FT , Ciccarelli O . Clinically isolated syndromes. Lancet Neurol 2012;11:157–169 22265211 10.1016/S1474-4422(11)70274-5

[7] Confavreux C , Vukusic C , Adeleine P . Early clinical predictors and progression of irreversible disability in multiple sclerosis: an amnesic process. Brain 2003;126:770–782. 12615637 10.1093/brain/awg081

[8] Fisniku LK , Brex PA , Altmann DR , et al. Disability and T2 MRI lesions: a 20‐year follow‐up of patients with relapse onset of multiple sclerosis. Brain 2008;131:808–817. 18234696 10.1093/brain/awm329

[9] Tintore M , Rovira A , Arrambide G , et al. Brainstem lesions in clinically isolated syndromes. Neurology 2010;75:1933–1938. 21098409 10.1212/WNL.0b013e3181feb26f

[10] Brownlee WJ , Altmann DR , Alves Da Mota P , et al. Association of asymptomatic spinal cord lesions and atrophy with disability 5 years after a clinically isolated syndrome. Mult Scler 2017;23:665–674. 27481210 10.1177/1352458516663034

[11] Miller DH , Ormerod IE , McDonald WI , et al. The early risk of multiple sclerosis after optic neuritis. J Neurol Neurosurg Psychiatry 1988;51:1569–1571. 3221224 10.1136/jnnp.51.12.1569 PMC1032775

[12] Miller DH , Ormerod IEC , Rudge P , et al. The early risk of multiple sclerosis following isolated acute syndromes of the brainstem and spinal cord. Ann Neurol 1989;26:635–639. 2817838 10.1002/ana.410260508

[13] Morrissey SP , Miller DH , Kendall BE , et al. The significance of brain magnetic resonance imaging abnormalities at presentation with clinically isolated syndromes suggestive of multiple sclerosis. Brain 1993;116:135–146. 8453454 10.1093/brain/116.1.135

[14] O'Riordan JI , Thompson AJ , Kingsley DPE , et al. The prognostic value of brain MRI in clinically isolated syndromes of the CNS. Brain 1998;121:495–503. 9549525 10.1093/brain/121.3.495

[15] Brex PA , Ciccarelli O , O'Riordan JI , et al. A longitudinal study of abnormalities on MRI and disability from multiple sclerosis. N Engl J Med 2002;346:158–164. 11796849 10.1056/NEJMoa011341

[16] Kurtzke JF . Rating neurologic impairment in multiple sclerosis: an expanded disability status scale (EDSS). Neurology 1983;33:1444–1452. 6685237 10.1212/wnl.33.11.1444

[17] Lechner‐Scott L , Kappos L , Hofman M , et al. Can the Expanded Disability Status Scale be assessed by telephone? Mult Scler 2003;9:154–159. 12708811 10.1191/1352458503ms884oa

[18] Fischer JS , Rudick RA , Cutter GR , Reingold SC . The Multiple Sclerosis Functional Composite Measure (MSFC): an integrated approach to MS clinical outcome assessment. National MS Society Clinical Outcomes Assessment Task Force. Mult Scler 1999;5:244–250. 10467383 10.1177/135245859900500409

[19] Langdon DW , Amato MP , Boringa J , et al. Recommendations for a brief international cognitive assessment for multiple sclerosis (BICAMS). Mult Scler 2012;18:891–898. 22190573 10.1177/1352458511431076 PMC3546642

[20] Polman CH , Reingold SC , Banwell B , et al. Diagnostic criteria for multiple sclerosis: 2010 revisions to the McDonald criteria. Ann Neurol 2011;69:292–302. 21387374 10.1002/ana.22366 PMC3084507

[21] Lublin, F , Reingold S . Defining the clinical course of multiple sclerosis: results of an international survey. National Multiple Sclerosis Society (USA) Advisory Committee on Clinical Trials of New Agents in Multiple Sclerosis. Neurology 1996;46:907–911. 8780061 10.1212/wnl.46.4.907

[22] Amato MP , Zipoli V , Goretti B , et al. Benign multiple sclerosis: cognitive, psychological and social aspects in a clinical cohort. J Neurol 2006;253:1054–1059. 16609810 10.1007/s00415-006-0161-8

[23] Ebner M , Chung KK , Prados F , et al. Volumetric reconstruction from printed films: enabling 30 year longitudinal analysis in MR neuroimaging. Neuroimage 2018;165:238–250. 29017867 10.1016/j.neuroimage.2017.09.056 PMC5737406

[24] Fedorov A , Beichel R , Kalpathy‐Cramer J , et al. 3D Slicer as an image computing platform for the quantitative imaging network. Magn Reson Imaging 2012;30:1323–1341. 22770690 10.1016/j.mri.2012.05.001 PMC3466397

[25] Stata [computer program] . Version 15. College Station, TX: StataCorp, 2017.

[26] Cree B , Gourraud PA , Oksenberg JR , et al. Long‐term evolution of multiple sclerosis disability in the treatment era. Ann Neurol 2016;80:499–510. 27464262 10.1002/ana.24747 PMC5105678

[27] Sayao A , Devonshire V , Tremlett H . Longitudinal follow‐up of “benign” multiple sclerosis at 20 years. Neurology 2007;68:496–501. 17296915 10.1212/01.wnl.0000253185.03943.66

[28] Tallantyre EC , Major PC , Atherton MJ , et al. How common is truly benign MS in a UK population? J Neurol Neurosurg Psychiatry 2018;0:1–7. 10.1136/jnnp-2018-318802 PMC6581074 30177509

[29] Tintore M , Rovira À , Río J , et al. Defining high, medium and low impact prognostic factors for developing multiple sclerosis. Brain 2015;138:1863–1874. 25902415 10.1093/brain/awv105

