# Supplementary material for: A 30‐Year Clinical and Magnetic Resonance Imaging Observational Study of Multiple Sclerosis and Clinically Isolated Syndromes
Source: Ann Neurol. 2019 Nov 22;87(1):63–74. doi: 10.1002/ana.25637 (PMC6973080; doi:10.1002/ana.25637)
Supplement: Supplementary file 1 — SUPPLEMENTARY TABLE 1 Early Univariable Predictors of 30‐Year Outcomes [file ANA-87-63-s001.docx]

SUPPLEMENTARY TABLE. Early Univariable Predictors of 30-Year Outcomes

| **Predictor** | | | **30-year EDSS ≤3.5 vs 30-year EDSS >3.5** | | | **SPMS by 30 years** | | | **Death related to MS by 30 years** | | |
| --- | --- | --- | --- | --- | --- | --- | --- | --- | --- | --- | --- |
|  |  |  | *n* | *Odds ratio*  *(95% CI)* | *P-value* | *n* | *Odds ratio*  *(95% CI)* | *P-value* | *n* | *Odds ratio*  *(95% CI)* | *P-value* |
| Demographic and clinical features | CIS type | TM vs brainstem | 107 | 0.61 (0.20, 1.87) | 0.383 | 119 | 0.62 (0.21, 1.82) | 0.385 | 119 | 0.27 (0.06, 1.21) | 0.087 |
|  |  | ON vs brainstem | 107 | 0.63 (0.23, 1.74) | 0.375 | 119 | 0.67 (0.26, 1.71) | 0.397 | 119 | 0.33 (0.10, 1.11) | 0.074 |
|  | Age at onset, OR per year | | 102 | 1.05 (1.00, 1.11) | 0.060 | 112 | 1.02 (0.97, 1.07) | 0.411 | 117 | 1.08 (1.00, 1.16) | 0.036 |
|  | Gender, male vs female | | 107 | 1.12 (0.50, 2.48) | 0.787 | 119 | 0.97 (0.45, 2.14) | 0.949 | 119 | 0.98 (0.33, 2.92) | 0.978 |
|  | Disease duration, OR per year | | 102 | 1.54 (0.59, 4.01) | 0.378 | 111 | 1.40 (0.69, 2.88) | 0.353 | 110 | 0.69 (0.10, 4.63) | 0.702 |
|  | No. relapses in first 5 years, 0/1/2/3+ | | 95 | 2.37 (1.54, 3.65) | <0.001 | 103 | 2.45 (1.61, 3.73) | <0.001 | 100 | 2.18 (1.26, 3.77) | 0.005 |
| EDSS | Baseline EDSS ≥2.5 vs <2.5 | | 100 | 1.89 (0.81, 4.42) | 0.140 | 110 | 2.57 (1.06, 6.20) | 0.036 | 108 | 4.00 (0.84, 19.05) | 0.082 |
|  | Nadir EDSS ≥2.5 vs <2.5 | | 105 | 6.44 (1.68, 24.77) | 0.007 | 115 | 2.55 (0.90, 7.25) | 0.079 | 111 | 3.12 (0.84, 11.69) | 0.090 |
|  | 5-year EDSS ≥2.5 vs <2.5 | | 105 | 15.61 (4.83, 50.48) | <0.001 | 109 | 9.43 (3.41, 26.06)* | <0.001 | 110 | 30.00 (6.12, 147.15) | <0.001 |
|  | EDSS change baseline to 5-year ≥0 vs <0 | | 100 | 8.84 (3.44, 22.76) | <0.001 | 103 | 4.98 (2.03, 12.19)* | <0.001 | 105 | 8.02 (2.05, 31.45) | 0.003 |
|  | EDSS change nadir to 5-year ≥2 vs <2 | | 105 | 31.03 (3.91, 246.49) | 0.001 | 109 | 40.70 (5.05, 328.17)* | <0.001 | 110 | 19.80 (5.34, 73.46) | <0.001 |
| Lesion counts | Total baseline lesion count, 0/1-3/4/10/11-20/21+ | | 103 | 1.84 (1.35, 2.52) | <0.001 | 114 | 1.86 (1.37, 2.52) | <0.001 | 108 | 2.03 (1.32, 3.12) | 0.001 |
|  | Total baseline lesion count, 1+ vs 0 | | 103 | 5.21 (1.91, 14.2) | 0.001 | 114 | 7.63 (2.47, 23.62) | <0.001 | 108 | 7.62 (0.96, 60.40) | 0.054 |
|  | Total 1-year lesion count, 0/1-4/5-15/16+ | | 86 | 2.51 (1.60, 3.93) | <0.001 | 96 | 2.67 (1.70, 4.17) | <0.001 | 92 | 2.48 (1.31, 4.68) | 0.005 |
|  | Total 1-year lesion count, 1+ vs 0 | | 86 | 9.26 (2.48, 34.51) | 0.001 | 96 | 12.87 (2.83, 58.58) | 0.001 | 92 | 5.60 (0.69, 45.19) | 0.106 |
|  | Total 5-year lesion count, 0/1-10/11-20/21-40/41+ | | 81 | 2.57 (1.69, 3.90) | <0.001 | 88 | 2.30 (1.57, 3.39) | <0.001 | 85 | 2.26 (1.27, 4.02) | 0.005 |
|  | Total 5-year lesion count, 0-10 vs 11+ | | 81 | 6.84 (2.38, 19.70) | <0.001 | 88 | 6.70 (2.25, 19.92) | 0.001 | 52 | See ^ below | <0.001 |
|  | Total lesion change baseline to 1-year, 1+ vs 0 | | 86 | 3.41 (1.38, 8.45) | 0.008 | 96 | 3.95 (1.59, 9.81) | 0.003 | 91 | 3.41 (0.89, 13.08) | 0.073 |
|  | Total lesion change baseline to 5-year, 0/1-9/10+ | | 81 | 5.90 (2.57, 13.53) | <0.001 | 88 | 4.79 (2.16, 10.63) | <0.001 | 85 | 4.70 (1.18, 18.72) | 0.028 |
| Lesion locations | Baseline PV lesion count, ≥1 vs 0 | | 97 | 3.91 (1.63, 9.36) | 0.002 | 107 | 4.48 (1.85, 10.88) | 0.001 | 102 | 6.38 (1.37, 29.77) | 0.018 |
|  | Baseline DWM lesion count, ≥1 vs 0 | | 97 | 4.67 (1.83, 11.92) | 0.001 | 107 | 7.65 (2.67, 21.92) | <0.001 | 102 | 4.80 (1.03, 22.47) | 0.046 |
|  | Baseline JC lesion count, ≥1 vs 0 | | 97 | 4.05 (1.41, 11.62) | 0.009 | 107 | 3.67 (1.41, 9.58) | 0.008 | 102 | 4.00 (1.28, 12.53) | 0.017 |
|  | Baseline IT lesion count, ≥1 vs 0 | | 96 | 12.41 (3.35, 45.98) | <0.001 | 106 | 20.27 (5.43, 75.65) | <0.001 | 101 | 4.67 (1.51, 14.41) | 0.007 |
|  | 1-year PV lesion count, 1+ vs 0 | | 82 | 3.69 (1.34, 10.16) | 0.11 | 92 | 5.41 (1.84, 15.94) | 0.002 | 87 | 3.52 (0.73, 16.94) | 0.116 |
|  | 1-year DWM lesion count, 1+ vs 0 | | 82 | 10.65 (2.85, 39.84) | <0.001 | 92 | 14.93 (3.27, 68.18) | <0.001 | 87 | 7.19 (0.89, 58.12) | 0.064 |
|  | 1-year JC lesion count, 1+ vs 0 | | 82 | 4.76 (1.70, 13.31) | 0.003 | 92 | 3.80 (1.48, 9.73) | 0.005 | 87 | 2.84 (0.88, 9.16) | 0.080 |
|  | 1-year IT lesion count, 1+ vs 0 | | 82 | 11.1 (3.32, 37.23) | <0.001 | 92 | 19.29 (5.67, 65.58) | <0.001 | 87 | 5.93 (1.75, 20.09) | 0.004 |
|  | 5-year PV lesion count, 1+ vs 0 | | 81 | 4.43 (1.73, 11.31) | 0.002 | 88 | 4.28 (1.69, 10.86) | 0.002 | 85 | 4.06 (1.02, 16.26) | 0.047 |
|  | 5-year DWM lesion count, 1+ vs 0 | | 81 | 7.75 (2.83, 21.23) | <0.001 | 88 | 6.43 (2.36, 17.49) | <0.001 | 85 | 5.43 (1.11, 26.52) | 0.037 |
|  | 5-year JC lesion count, 1+ vs 0 | | 81 | 6.00 (2.26, 15.93) | <0.001 | 88 | 5.40 (2.05, 14.23) | 0.001 | 85 | 12.62 (1.55, 102.85) | 0.018 |
|  | 5-year IT lesion count, 1+ vs 0 | | 78 | 8.85 (3.07, 25.50) | <0.001 | 85 | 8.21 (3.01, 22.38) | <0.001 | 82 | 6.13 (1.51, 24.83) | 0.011 |

TM= transverse myelitis; ON= optic neuritis; PV= periventricular; DWM= deep white matter; JC= juxta-cortical; IT= infra-tentorial. For all ordinal variables, ORs apply to per adjacent category.

*excluding three subjects who had already developed secondary progression by 5 years

^OR not estimated due to zero deaths in the 0-10 lesion group
